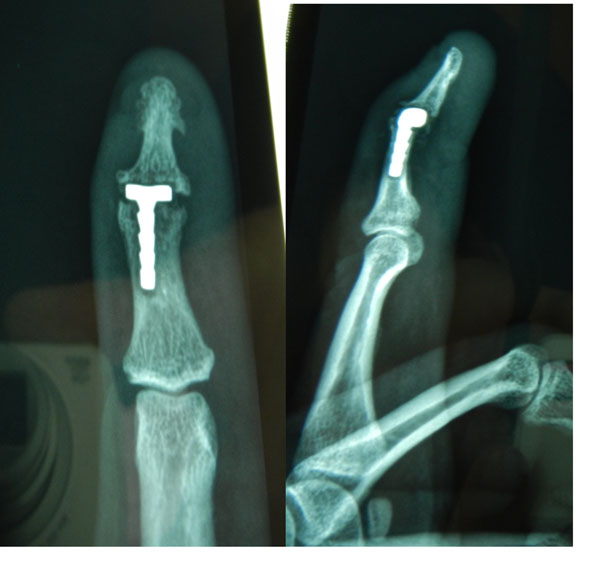# Distal interphalangeal hemiarthroplasty (ADIP): preliminary series of 14 cases

**DOI:** 10.1186/1753-6561-9-S3-A88

**Published:** 2015-05-19

**Authors:** Pierre Jean Regnard

**Affiliations:** 1 SOS Mains Dijon, Point Medical, Dijon, 21000, France

## Introduction

The DIP Argo hemiprosthesis (ADIP) was designed in 2012 with the idea of replacing the destroyed / arthritic head of the middle phalanx (P2) and was implanted in 2013 for the first time.Basically the idea was to achieve coverage of P2 as opposed to P3 (distal phalanx), because the last phalanx of the digit is so thin, and the joint surface often so destroyed that the fixation of the distal part is deemed not feasible.

The implant comprises part of thesurface of a cylinder that covers only the articular part of the middle phalanx, and is fixed to a stem 8mm long and very narrow, inserted into the medullary cavity.The stem is coated with Hydroxyapatite(HAP) and one size fits all.

## Methods

A midline dorsal approach is used and the extensor tendon divided longitudinally as routinely done. 2mm off the head of P2 is removed, then a midline 1.2mmK-wire is fixed as guide in the medullary cavity.

With a special rasp it’s possible simultaneously to flatten part of the head of the middle phalanx, in order to prepare the site of the stem of the ADIP and to smoothen the proximal surface of the distal phalanx too, since it is often damaged.

The trial implant is then inserted, and more resection is carried out if necessary (especially when tested in flexion).The ADIP prosthesis is inserted, impacted in its definitive position, and finally the mobility of the DIP is tested.If necessary the volar portion of the condyle of P2 is resected as a chamfer cut.Repair of the extensor system is performed just before skin closure.

## Results

Our series is very small with a relatively short maximal follow-up of 18 months.The most frequent indication was painful osteoarthritis of the DIPJ. In 13 cases, pain disappeared, while mobility increased to 30° in 9 cases, and less than 15° in 5 patients.An extension lag (of less than 10°) of the distal phalanx was seen in 2 instances, which was really a lack of hyperxetension.No lateral instability was seen.

## Discussion and conclusion

The fact that the pain disappears is very interesting, and can be explained to some extent by the possible denervation caused by the dorsal approach, similar to other prostheses being inserted with the same approach.The recovery time is determined by the period needed for rehabilitation of the repaired extensor system. Mobility is limited initially, but sufficient for grasp without pain, which makes this the reason of the choice for patients instead of fusion.

It is still too early to predict the ‘natural history’ of this prosthesis, but for those on the 18 months’ follow-up, the fixation seems very good,with good density of the bone close to the stem, and against the implant himself.No loosening was seen.

Caveat: In case of a bad result it is easy to perform fusion as before.

**Figure 1:**